# Two Carya Species, *Carya hunanensis* and *Carya illinoinensis*, Used as Rootstocks Point to Improvements in the Heat Resistance of *Carya cathayensis*

**DOI:** 10.3390/plants13141967

**Published:** 2024-07-18

**Authors:** Shanxia Huang, Yanxia Xu, Xueqin Li, Boyu Ye, Songheng Jin

**Affiliations:** Jiyang College, Zhejiang A&F University, Zhuji 311800, China; shanxia_huang@126.com (S.H.); lxqin@zafu.edu.cn (X.L.); yby2019602041053@163.com (B.Y.)

**Keywords:** grafting, phytohormone, transcriptome, heat stress, Chinese hickory

## Abstract

Grafting as a crucial horticultural technique has been widely used in the cultivation of *Carya cathayensis* (Chinese hickory), which is a unique and important economic tree in the northeast of Zhejiang Province and the south of Anhui Province. However, the existing literature lacks research on the potential impact of various rootstocks on the thermal tolerance of Chinese hickory. The objectives of this study were to evaluate heat tolerance in four distinct groups of Chinese hickory, including *C. cathayensis* grafted onto *Carya hunanensis* and *Carya illinoinensis*, one self-grafted group (*C. cathayensis* grafted onto *C. cathayensis*), and one non-grafted group (*C. cathayensis*). We examined photosynthesis parameters, phytohormones, and differentially expressed genes in the four various hickory groups subjected to 25 °C, 35 °C, and 40 °C heat stress (HS). The results demonstrated that grafting onto *C. hunanensis* and *C. illinoinensis* exhibited a higher net photosynthetic rate and stomatal conductance, lower intercellular CO_2_ concentration, and smaller changes in plant hormone content compared to self-grafted and non-grafted group under HS. The transcriptome results revealed that the majority of differentially expressed genes (DEGs) associated with photosynthetic pathways exhibited downregulation under HS, while the degree of variation in grafted groups using *C. hunanensis* and *C. illinoinensis* as rootstocks was comparatively lower than that observed in self-grafted and non-grafted groups. The alteration in the expression patterns of DEGs involved in plant hormone synthesis and metabolism under HS corresponded to changes in plant hormone contents. Overall, Chinese hickory grafted onto *C. hunanensis* and *C. illinoinensis* exhibited enhanced resistance to high-temperature stress at the juvenile stage.

## 1. Introduction

With the exacerbation of the greenhouse effect, global temperatures have experienced a rapid increase. In recent years, there has been a significant rise in the frequency of short-term extreme high temperatures, making high temperature an increasingly prominent abiotic stress factor that restricts plant growth and development during summer [1,2]. Among various abiotic stresses, plants are particularly susceptible to heat stress (HS), which is often accompanied by photo-oxidative stress. HS not only severely hampers plant growth and development through damage to photosynthetic components but also disrupts redox balance and alters the expression of stress resistance genes and proteins [3]. Through evolutionary processes, plants have developed intricate mechanisms for heat stress response (HSR) to mitigate the detrimental effects caused by HS.

The mechanisms of HSR can regulate a range of biological activities, including osmoprotection, antioxidant activity, hormonal signaling, metabolite synthesis, and induction of heat shock proteins (HSPs) [4]. During HS, proline and other osmotic regulatory substances are induced and accumulated to protect the plant cell membrane from thermal damage [5]. Moreover, numerous antioxidants such as peroxidase (POD) and superoxide dismutase (SOD) rapidly increase to eliminate excessive reactive oxygen species (ROS) and enhance plant resistance to HS [6]. Furthermore, transcription factors serve as primary regulators in this process; among them are the heat shock transcription factors (HSFs), which activate the expression of HSP genes [7,8]. Studies have reported that heat stress promotes the accumulation of HSPs acting as molecular chaperones that safeguard cells against protein folding and degradation caused by high temperatures [9]. Accumulating evidence suggests that HSPs play crucial roles in conferring heat tolerance, with specific HSPs being causally involved in acquiring the ability to withstand heat stress [10].

Plant hormones are essential messenger molecules in higher plants, which mediate plant growth, development, and responses to diverse biotic and abiotic stresses. To date, all major hormones such as abscisic acid (IAA), auxin, gibberellins (GAs), cytokinins (CTKs), salicylic acid (SA), jasmonic acid (JA), ethylene (ETH), and brassinosteroids (BRs) have been reported to play critical roles in the response of plants to heat stress [11,12,13,14,15,16]. Under HS, plant hormones play a pivotal role in orchestrating numerous physiological processes. These include fostering root development, modulating photosynthesis, regulating stomatal aperture, facilitating the accumulation of osmolytes, aiding in pollen development, and mitigating the accumulation of reactive oxygen species (ROS). Collectively, these hormonal activities help plants to successfully adapt to and thrive in high-temperature environments [17].

Chinese hickory (*Carya cathayensis*), renowned for its edible nuts, serves as a significant economic tree in the northeastern region of Zhejiang Province and southern area of Anhui Province. The seeds of Chinese hickory contain valuable chemical components such as polyunsaturated fatty acids, phenolics, and flavones [18], which possess both nutritional and cytotoxic properties. Grafting is an ancient and efficient method, which has been widely used in agriculture, horticulture and various tree species. Grafting provides a flexible toolkit to modify and enhance plant traits, improve disease resistance, and produce better crop yields [19]. Grafting has been used in the Chinese hickory industry for it is an effective technique in reducing the juvenile stage duration of Chinese hickory while simultaneously enhancing plant yield, product quality, and overall health [20]. Zhou et al. [21] found that Chinese hickory grafted onto *Carya illinoinensis* and *Carya hunanensis* showed more resistance against *B. dothidea* trunk canker disease. Numerous studies have indicated that grafting can confer resistance against various biotic and abiotic stresses, such as water stress, temperature fluctuations, salinity levels, and heavy metal exposure, including HS [19,22,23,24]. For instance, grafted tomato plants exhibited increased resistance to heat stress compared to non-grafted plants by displaying higher antioxidant enzyme activities along with lower hydrogen peroxide (H_2_O_2_) concentrations. Additionally observed were improved chlorophyll fluorescence levels and reduced electrolyte leakage in grafted plants [25,26]. Similarly, grafted pepper [27] and cucumber [28] plants displayed elevated chlorophyll contents along with maximum quantum yields of PSII and Fv/Fm when compared to non-grafted or self-grafted counterparts. Moreover, previous research has demonstrated that grafting onto heat-tolerant rootstocks significantly enhances the heat resistance capabilities of Rosa chinensis ‘Old Blush’ [29] as well as cucumber varieties [28]. Gisbert-Mullor et al. [27] reported that pepper grafted onto appropriate rootstocks exhibited higher relative growth rates along with improved Fv/Fm values and leaf areas under high-temperature stress conditions.

Currently, different plants have been studied to observe the effects of grafting on various plant characteristics, such as water potential, leaf gas exchange, photochemical efficiency of photosystem II, and biosynthesis of plant hormones [16,30,31,32]. Moreover, previous research has highlighted the beneficial role played by phytohormones like IAA, CTK, and GA [16], as well as specific gene families including *AUX/LAX* family genes [33] and *PIN* family genes [34], in the process of Chinese hickory grafting. However, there is a lack of research in the existing literature regarding the potential impact of different rootstocks on the thermal tolerance of Chinese hickory.

In order to investigate the impact of different rootstocks on the heat resistance of grafted Chinese hickory plants, with a particular focus on the influence of photosynthesis and phytohormones, we conducted experiments using Chinese hickory grafted onto *C. hunanensis*, *C. illinoinensis* and *C. cathayensis*, and non-grafted plants. Transcriptome sequencing was performed on four Chinese hickory groups at 0 days and 4 days after exposure to heat treatment. Additionally, gas exchange parameters were measured, and phytohormone contents were detected. Differentially expressed genes (DEGs), specifically those associated with photosynthesis, heat stress response (HSR), and phytohormones were identified through transcriptome analysis. The objective of this study is to study the effects of grafting on the heat resistance of Chinese hickory and to provide a new idea for the breeding of heat-resistant varieties of Chinese hickory.

## 2. Results

### 2.1. Effects of Heat Stress on Gas Exchange and Chlorophyll Fluorescence Parameters

**Figure 1:**
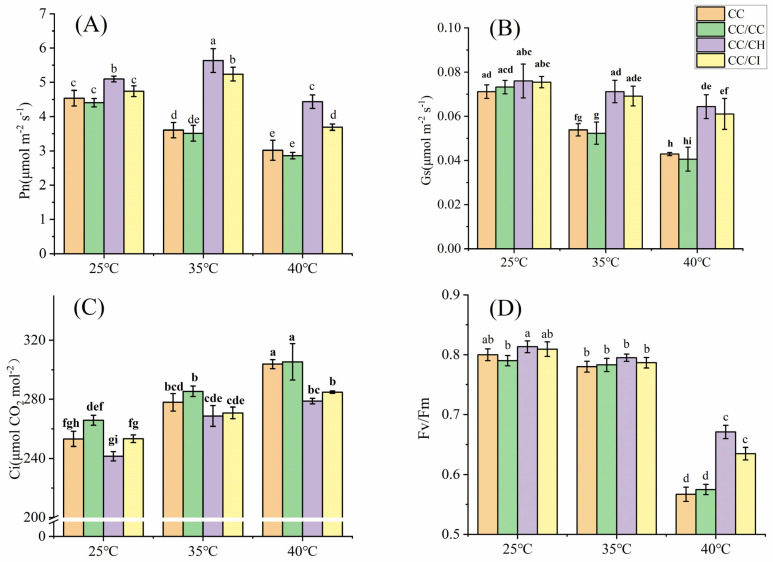
Effects of heat stress on gas exchange and Fv/Fm of grafted *Carya cathayensis* leaves under heat stress. CC: non-grafted *Carya cathayensis*, CC/CC: self-grafted *Carya cathayensis*, CC/CH: *Carya cathayensis* grafted onto *Carya hunanensis*. CC/CI: *Carya cathayensis* grafted onto *Carya illinoinensis.* Fv/Fm: maximum efficiency of PSII. (**A**): Changes in net photosynthetic rate under heat stress. (**B**): Changes in stomatal conductance under heat stress. (**C**): Changes in intercellular CO_2_ concentration under heat stress. (**D**): Changes in Fv/Fm under heat stress. Means (*n* = 3) followed by the same letter are not significantly different at *p* < 0.05 (Duncan’s test).

**Figure 2:**
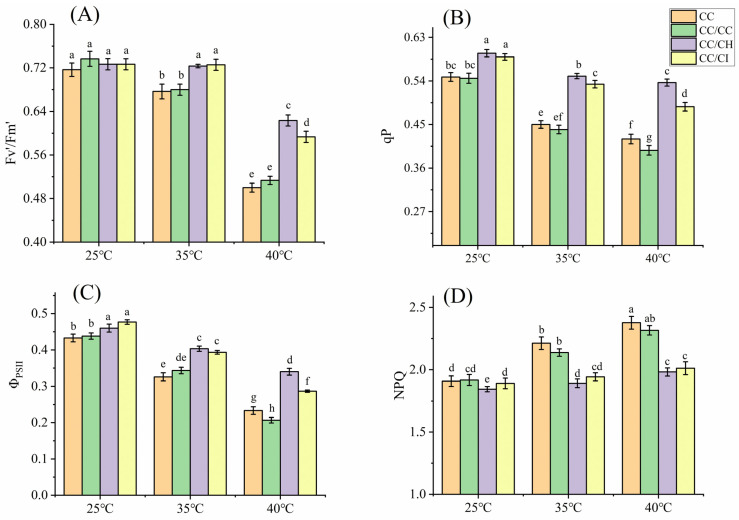
Effects of heat stress on Fv’/Fm’, qP, Φ_PSII_ and NPQ in Chinese hickory leaves. CC: non-grafted *Carya cathayensis*, CC/CC: self-grafted *Carya cathayensis*, CC/CH: *Carya cathayensis* grafted onto *Carya hunanensis*. CC/CI: *Carya cathayensis* grafted onto *Carya illinoinensis.* Fv’/Fm’: effective quantum yield of PSII photochemistry. Φ_PSII_: the efficiency of PSII. qP: photochemical. NPQ: nonphotochemical quenching. (**A**): Effects of heat stress on Fv’/Fm’ in Chinese hickory. (**B**): Effects of heat stress on qP in Chinese hickory. (**C**): Effects of heat stress on Φ_PSII_ in Chinese hickory. (**D**): Effects of heat stress on NPQ in Chinese hickory leaves. Means (n = 3) followed by the same letter are not significantly different at *p* < 0.05 (Duncan’s test).

Under mild heat stress (35 °C), the photosynthetic rate (Pn) of *C. cathayensis* grafted onto *C. hunanensis* (CC/CH) and *C. cathayensis* grafted onto *C. illinoinensis* (CC/CI) significantly increased, while the Pn of self-grafted *C. cathayensis* (CC/CC) and non-grafted *C. cathayensis* (CC) decreased. At 40 °C, the Pn of CC/CH and CC/CI maintained a relatively higher level with a lower decrease rate compared to that of CC/CC and CC (Figure 1A). The Pn of CC, CC/CC, CC/CH, and CC/CI decreased by 33.5%, 35.0%, 12.9%, and 22.2%, respectively, compared to the treatment at 25 °C. The stomatal conductance (Gs) decreased under HS as the stress temperature increased, with larger decreases observed for CC/CC and CC compared to CC/CH and CC/CI. At 40 °C, the Gs of CC, CC/CC, CC/CH, and CC/CI decreased by 39.7%, 44.6%, 15.3%, and 19.1%, respectively, compared to the treatment at 25 °C. The impact of HS on the Gs was much higher in CC/CC and CC than that in CC/CH and CC/CI. (Figure 1B). The intercellular CO_2_ concentration (Ci) of CC, CC/CC, CC/CH, and CC/CI exhibited a gradual increase with the rise in temperature (Figure 1C), showing increments of 27.4%, 27.0%, 19.8%, and 18.1% at 40 °C compared to 25 °C, respectively. Under heat stress, the maximum efficiency of PSII (Fv/Fm), effective quantum yield of PSII photochemistry (Fv’/Fm’), the efficiency of PSII (Φ_PSII_) and photochemical (qP) of the four Chinese hickory groups all showed a decreased trend with increased temperature, while nonphotochemical quenching (NPQ) increased with increased temperature. In this study, the variation in these parameters in CC/CH and CC/CI is obviously greater than that in CC and CC/CC (Figure 1D and Figure 2).

### 2.2. Effects of Heat Stress on Hormone Contents

**Figure 3:**
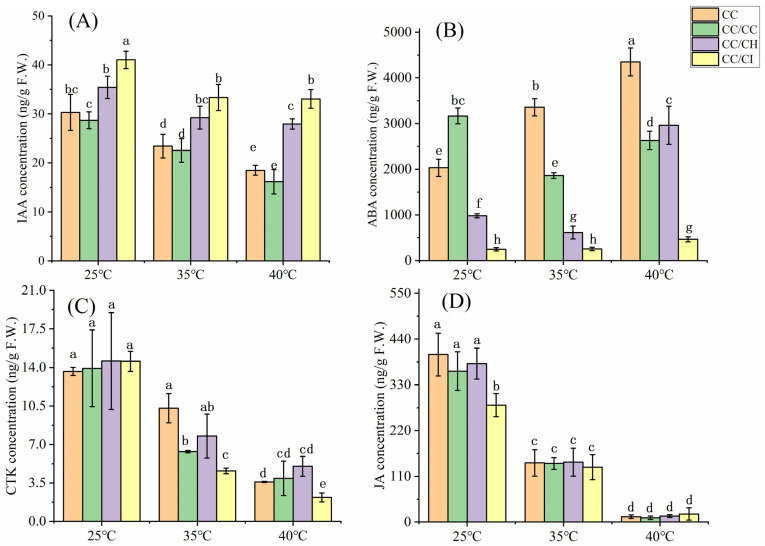
Concentration changes in different phytohormones in grafted *Carya cathayensis* under heat stress. CC: non-grafted *Carya cathayensis*, CC/CC: self-grafted *Carya cathayensis*, CC/CH: *Carya cathayensis* grafted onto *Carya hunanensis*. CC/CI: *Carya cathayensis* grafted onto *Carya illinoinensis*. (**A**): IAA content in grafted *Carya cathayensis* under heat stress. (**B**): ABA content in grafted *Carya cathayensis* under heat stress. (**C**): CTK content in grafted *Carya cathayensis* under heat stress. (**D**): JA content in grafted *Carya cathayensis* under heat stress. Means (n = 3) followed by the same letter are not significantly different at *p* < 0.05 (Duncan’s test).

The IAA, CTK, and JA contents exhibited a declining trend under HS conditions at both 35 °C and 40 °C. At 35 °C, the IAA content in CC, CC/CC, CC/CH, and CC/CI decreased by 22.7%, 21.4%, 17.5%, and 18.7%, respectively, whereas at 40 °C, the IAA content decreased by 38.9%, 43.7%, 21.1%, and 19.4% in CC, CC/CC, CC/CH, and CC/CI, respectively (Figure 3A). Obviously, there was a significantly greater reduction in IAA levels observed in the CC and CC/CC groups compared to the CC/CH and CC/CI groups following heat treatment at 40 °C, as opposed to 25 °C. In terms of CTKs, the concentrations exhibited a decline of over 60% across all four Chinese hickory groups at 40 °C, while these decreased only in CC and CC/CH at 35 °C (Figure 3C). Notably, among these groups, the CTK content of CC/CH was the highest among the four groups, which also showed the smallest decrease in CTK content at 40 °C compared to 25 °C. The decrease in JA was more pronounced at 35 °C compared to IAA and CTKs, with a sharp decline of over 90% observed at 40 °C. At 40 °C, the ABA levels exhibited an increase in all the Chinese hickory groups except in CC/CC (Figure 3B). However, at 35 °C, the ABA contents decreased in CC/CC, CC/CH and CC/CI but increased in CC. Furthermore, the ABA increase was greater in CC/CH and CC/CI at 40°C when compared to the control temperature of 25 °C.

### 2.3. Identification of Differentially Expressed Genes

After the quality control step, an average of 6.4 Gb of clean data was obtained from each sample, representing over 93.8% of the original data (Table S2). The percentage of Q30 bases ranged from 93.69% to 94.87% (Table S2), indicating reliable sequencing quality. The clean reads were then aligned and annotated to the *C. cathayensis* reference genome (http://gigadb.org/dataset/view/id/100571/Sample_page/2 (accessed on 12 July 2024)) [18] using HISAT2 (http://ccb.jhu.edu/software/hisat2/index.shtml (accessed on 12 July 2024)). More than 90% of the clean reads were successfully aligned to the reference genome (Table S2). Principal component analysis revealed that PC1 and PC2 accounted for 50% and 24% of the variance, respectively (Figure S1), indicating high reproducibility of the transcriptome sequencing data, thus making them suitable for subsequent analysis. Figure S2 shows the correlation analysis of the samples. The number of differentially expressed genes (DEGs) (*p* < 0.05 and|log2 (fold change)| ≥ +1.0) for each comparison is presented in Table 1. A total of 658 DEGs (345 upregulated and 313 downregulated), 3816 DEGs (2442 upregulated and 1372 downregulated), 3691 DEGs (1587 upregulated and 2104 downregulated) and 3501 DEGs (1435 upregulated and 2066 downregulated) were identified in the comparison between 35-CC25-CC, 35-CC/CH and 25-CC/CH, 35-CC/CI and 25-CC/CI and 35-CC/CC and 25-CC/CC.

Under conditions of 40 °C, 3745 DEGs (2015 upregulated and 1730 downregulated), 7058 DEGs (3475 upregulated and 3585 downregulated), 6887 DEGs (3324 upregulated and 3563 downregulated), 5958 DEGs (3446 upregulated and 2512 downregulated) were identified in 40-CC vs. 25-CC, 40-CC/CH vs. 25-CC/CH, 40-CC/CI vs. 25-CC/CI and 40-CC/CC and 25-CC/CC. Overall, there were more DEGs in the 40 °C treatments than in the 35 °C treatments.

### 2.4. GO Enrichment and KEGG Pathway Analysis of Differentially Expressed Genes

**Figure 4:**
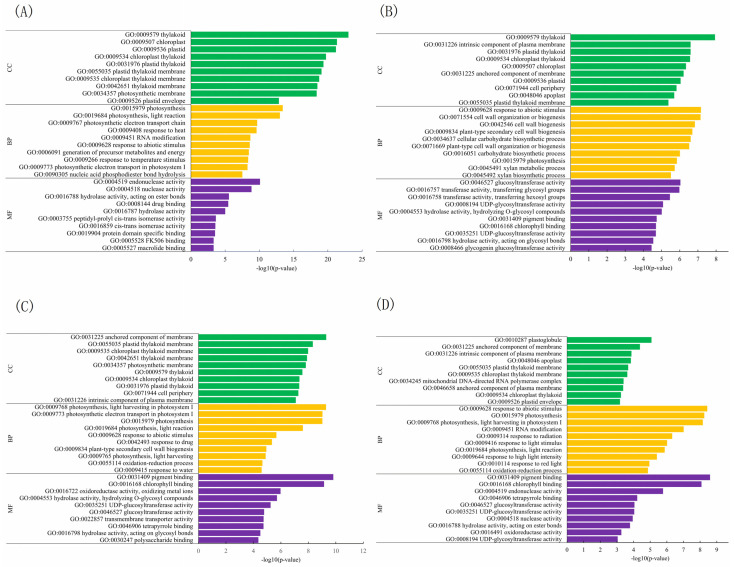
The top 10 enriched GO terms in four comparisons at 40 °C. BP: biological process; CC: component category; MF: molecular function. Numbers 25, 40 indicate grafted plants treated at 25 °C and 40 °C for 4 days, respectively. CC: non-grafted *Carya cathayensis*, CC/CC: self-grafted *Carya cathayensis*, CC/CH: *Carya cathayensis* grafted onto *Carya hunanensis*. CC/CI: *Carya cathayensis* grafted onto *Carya illinoinensis.* (**A**): 40-CC vs. 25-CC, (**B**): 40-CC/CC vs. 25-CC/CC, (**C**): 40-CC/CI vs. 25-CC/CI, (**D**): 40-CC/CH vs. 25-CC/CH. The default parameters for screening genes with differential expression with a *p*-value < 0.05 and a fold difference greater than 2.

The functions of DEGs were explored by utilizing public databases (GO, KEGG, KO, Swiss-Prot, and eggNOG). The top 10 enriched gene ontology (GO) terms in three categories—biological process, cellular component, and molecular function—at temperatures of 35 °C and 40 °C are presented in Figure 4 and Figure S3, and Table S4. After a 4-day treatment the enriched terms of DEGs varied across different comparisons.

**Figure 5:**
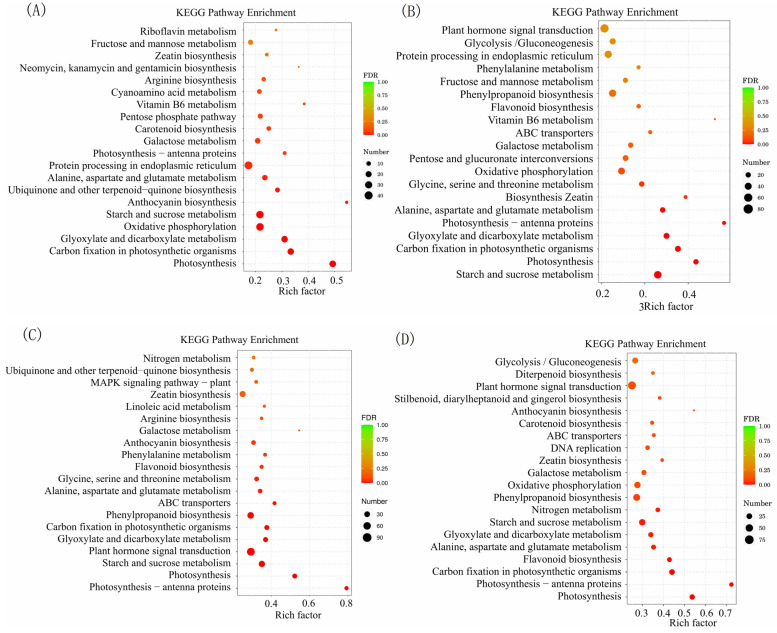
The top 20 enriched KEGG pathways in four comparisons of grafted *Carya cathayensis* exposure 40 °C treatments. (**A**): 40-CC vs. 25-CC, (**B**): 40-CC/CC vs. 25-CC/CC, (**C**): 40-CC/CH vs. 25-CC/CH, (**D**): 40-CC/CI vs. 25-CC/CI; Numbers 25 and 40 indicate grafted plants treated at 25 °C and 40 °C for 4 days, respectively. CC: non-grafted *Carya cathayensis*, CC/CC: self-grafted *Carya cathayensis*, CC/CH: *Carya cathayensis* grafted onto *Carya hunanensis*. CC/CI: *Carya cathayensis* grafted onto *Carya illinoinensis.* The default parameters for screening genes with differential expression with a *p*-value < 0.05 and a fold difference greater than 2.

The top 20 enriched KEGG terms for each comparison are presented in Figure 5 and Figure S4, and Table S5. The results of our study revealed a significant number of DEGs associated with crucial biological pathways, such as photosynthesis and plant hormone signal transduction. These findings suggest that these genes may play a pivotal role in the process of HSR in Chinese hickory.

### 2.5. Key Differentially Expressed Genes in Response to Heat Stress

According to our transcriptome analysis, genes associated with plant hormone signal transduction, transcription factors, heat shock proteins, and photosynthesis were prominently implicated in the HSR. We specifically focus on these genes in the subsequent analysis. Overall, there was a higher number of DEGs and more significant differences in gene expression observed under the 40 °C treatments. Therefore, only the results from the 40 °C treatments are presented in this analysis. The results from the 35 °C treatments can be found in Table S6.

#### 2.5.1. DEGs Involved in Photosynthesis under Heat Stress

**Figure 6:**
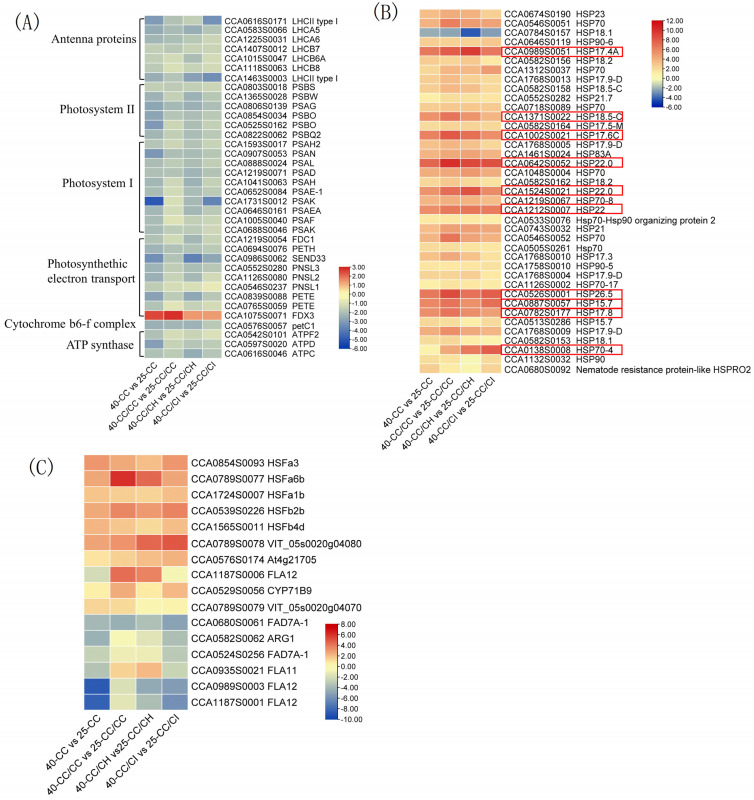
Heat map of DEGs related to HSR in the four grafted *Carya cathayensis* comparisons following exposure to 40 °C treatments. Numbers 25 and 40 indicate grafted plants treated with 25 °C and 40 °C for 4 days, respectively. CC: non-grafted *Carya cathayensis*, CC/CC: self-grafted *Carya cathayensis*, CC/CH: *Carya cathayensis* grafted onto *Carya hunanensis*. CC/CI: *Carya cathayensis* grafted onto *Carya illinoinensis.* The numbers on the different colored bars are log2|fold-change|values. (**A**): Heat map of DEGs related to photosynthesis in the four comparisons. (**B**): Heat map of DEGs related to HSPs in the four comparisons. (**C**): Heat map of DEGs related to HSF in the four comparisons. Genes in red box in Figure 6B were the genes with log2|fold-change| > 5 in more than two of the four comparations.

Overall, a greater number of DEGs involved in photosynthesis were identified at 40 °C compared to 35 °C in all the comparisons. Interestingly, the majority of these DEGs exhibited downregulation in this study, with their expression levels significantly more suppressed at 40 °C compared to 35 °C. At 40 °C, a total of 38 DEGs associated with photosynthesis were identified. Among them, there were 11 DEGs related to photosystem I, 9 DEGs related to photosynthetic electron transport, 7 DEGs related to antenna proteins, 7 DEGs related to photosystem II, 3 DEGs related to F-type ATPase, and 1 DEG related to the cytochrome b6f complex (Figure 6A). There were more DEGs found in CC/CH and CC/CI than in CC/CC and CC.

#### 2.5.2. Heat Shock Proteins Activated by HS

HSPs are a group of cellular proteins that are synthesized in response to stress-related stimulation. Figure 6B illustrates the differential expression of HSP genes under various heat treatments. We identified 27 differentially expressed HSPs at 35 °C and 39 differentially expressed HSPs at 40 °C, with 36 showing significant differential expression across all four comparisons. Overall, the expression levels of HSPs were higher under the 40 °C treatments compared to the 35°C treatments. Among the differentially expressed HSP genes, there were 10 genes with fold change values exceeding 30; most of them belonged to the small HSP family, including *HSP17.4A* (17.4 kDa class I heat shock protein), *HSP22.0* (22.0 kDa class IV heat shock protein), *HSP18.5-C* (18.5 kDa class I heat shock protein), and *HSP17.6C* (17.6 kDa class I heat shock protein 3). Additionally, nine genes exhibited fold change values ranging from 10 to 30, comprising five members of the HSP70 family, three sHSP family genes (*HSP17.9-D*, *HSP21*, and *HSP17.9-D*), and one instance of *HSP83A* gene expression alteration. Furthermore, all differentially expressed HSP genes demonstrated upregulation except for one specific case involving the 18.1 kDa class I heat shock protein (*HPS18*) (Figure 6B).

#### 2.5.3. Differentially Expressed Transcription Factors under HS

In this study, more than 50 TF family genes were annotated. Among them, ERF, bHLH, MYB-related, NAC, MYB, C2H2, and WRKY were identified as the top transcription factor families with the highest number of DEGs (Table S3). Additionally, HSFs were found to be the most significant class among heat stress response transcription factors. As depicted in Figure 6C, a majority of differentially expressed HSF genes exhibited upregulation. In the 40-CC vs. 25-CC, 40-CC/CC vs. 25-CC/CC, 40-CC/CI vs. 25-CC/CI, and 40-CC/CH vs. 25-CC/CH comparisons, there were 30 (13 upregulated and 17 downregulated), 43 (25 upregulated and 18 downregulated), 50 (27 upregulated and 23 downregulated), and 41 (22 upregulated and 19 downregulated) significantly DEGs in HSFs., respectively (Table S3). Among these DEGs, *HFsa1b*, *HFsa6b*, *HFs b2b*, *HFs b4d*, and *VIT_05s0020g04080* were significantly upregulated in all four comparisons; however, *FAD7A-1* was observed to be downregulated.

#### 2.5.4. DEGs Involved in Hormones under Heat Stress

**Figure 7:**
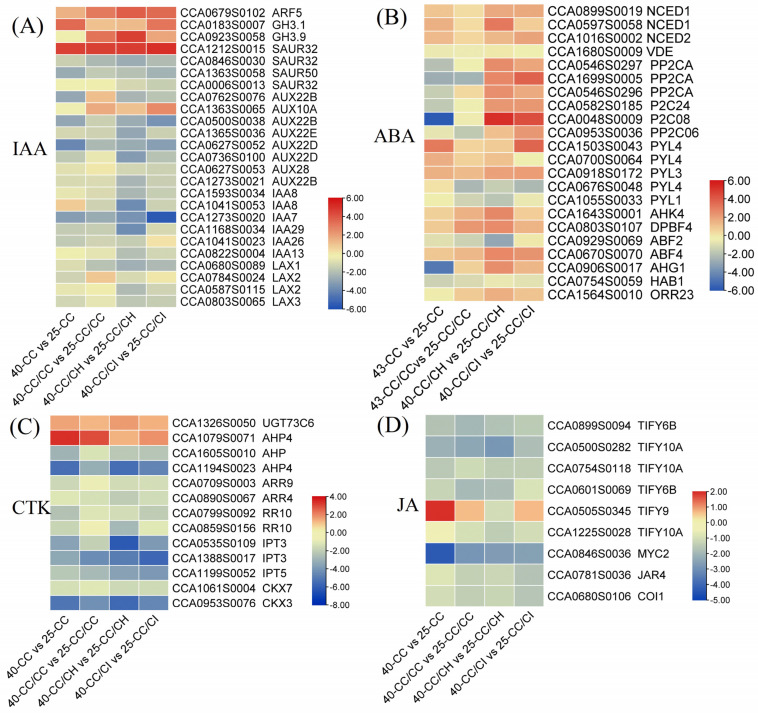
Heat map depicting the result of differential expression genes related to different hormones in the four grafted *Carya cathayensis* comparisons after 40 °C treatments. Numbers 25 and 40 indicate grafted plants treated with 25 °C and 40 °C for 4 days, respectively. CC: non-grafted *Carya cathayensis*, CC/CC: self-grafted *Carya cathayensis*, CC/CH: *Carya cathayensis* grafted onto *Carya hunanensis*. CC/CI: *Carya cathayensis* grafted onto *Carya illinoinensis.* The numbers on the different colored bars are log2fold-change values. (**A**): Heat map of differential expression genes related to auxin. (**B**): Heat map of differential expression genes related to abscisic acid. (**C**): Heat map of differential expression genes related to cytokinin. (**D**): Heat map of differential expression genes related to jasmonic acid.

**Figure 8:**
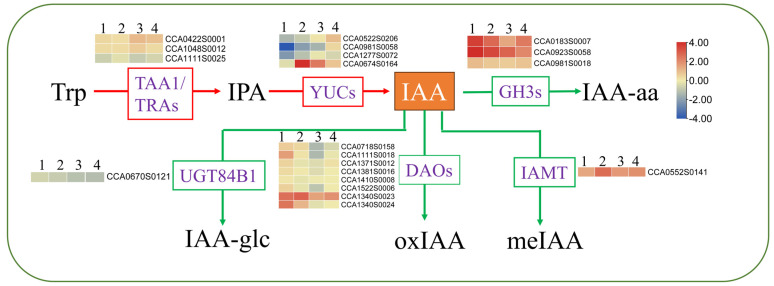
The primary routes for auxin biosynthesis and inactivation. 1. 40-CC vs. 25-CC; 2. 40-CC/CC vs. 25-CC/CC; 3. 40-CC/CH vs. 25-CC/CH; 4. 40-CC/CI vs. 25-CC/CI., precursor l-tryptophan (l-Trp), indole-3-pyruvic acid (IPA), Indole-3-acetic acid (IAA), TRYPTOPHAN AMINOTRANSFERASE OF ARABIDOPSIS (TAA), TAA1-RELATED proteins (TARs), flavin-containing monooxygenase (YUC) GRETCHEN HAGEN3 (GH3), IAA CARBOXYMETHYLTRANSFERASE1 (IAMT1), DIOXYGENASE FOR AUXIN OXIDATION (DAO), ester-linked IAA (IAA-glc), amide-linked IAA (IAA-aa), and methyl IAA (meIAA). The numbers on the different colored bars are log2fold-change values.

We identified 65 DEGs associated with the “plant hormone signal transduction” pathway, which exhibited enrichment in various phytohormone signal transduction clades, including IAA, ABA, CTKs, and JA. The most significantly enriched signal pathways were IAA, ABA, and CTK, with 28, 18, and 13 DEGs annotated to them, respectively. Among the DEGs associated with IAA, eight AUX (AUXIN) genes were found to be downregulated compared to 25 °C, and their expression levels were lower at 40 °C than at 35 °C. In contrast, genes of small auxin upregulated RNA (*SAUR32*; CCA1212S0015), indole-3-acetic acid-amido synthetase 3.9 (*GH3.9*; CCA0923S0058), and auxin response factor 5 (*ARF5*, CCA0679S0102) were found to be significantly upregulated. In addition, we identified 20 genes related to IAA synthesis and metabolism, and 4 ABA synthesizing genes (Figure 7B and Figure 8).

In addition, the fold change values of most DEGs were higher in CC/CH and CC/CI than in CC and CC/CC. Among these, *SAUR32* (CCA1212S0015) and *ARF5* (CCA0679S0102) exhibited log2 |fold change| values ranging from 3 to 6. Unlike auxin signaling-related DEGs, genes associated with CTKs and JA were all downregulated, while those related to ABA were found to be upregulated in response to heat stress. Interestingly, certain genes showed significant downregulation in CC and CC/CC but displayed significant upregulation in CC/CH and CC/CI compared to the 25 °C treatments, for example, protein phosphatase 2CA (*PP2CA*; CCA1699S0005), (*probable protein phosphatase 2C24* (*P2C24*; CCA0582S0185), and *two-component response regulator 23* (*ORR23*; CCA1564S0010) (Figure 7).

### 2.6. Validation of Transcription Data Using qRT-PCR

**Figure 9:**
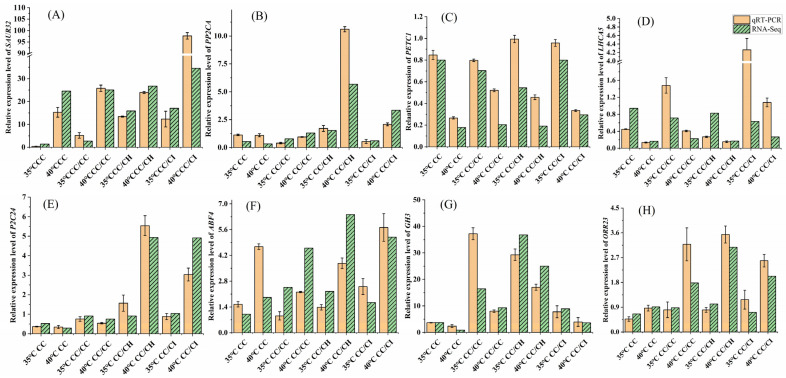
qRT-PCR confirmation of the DEGs identified by the transcriptome analysis. The relative expression level of each gene was expressed as the fold change. Actin was used as the reference gene. 35 °C CC means CC treated at 35 °C; 40 °C. CC means CC treated at 40 °C, and so on. (**A**): relative expression level of *SAUR32*, (**B**): relative expression level of *PP2CA*, (**C**): relative expression level of *PETC1*, (**D**): relative expression level of *LHCA5*, (**E**): relative expression level of *P2C24*, (**F**): relative expression level of *ABF4*, (**G**): relative expression level of *GH3*, (**H**): relative expression level of *ORR23*. The qRT-PCR data of each sample with three replicates.

To validate the accuracy of the RNA-seq data, we selected a total of eight genes for further quantitative RT-PCR (qRT-PCR) analysis. Overall, the expression patterns observed via qRT-PCR for these eight genes were consistent with the RNA-Seq data (Figure 9), indicating a high level of reliability in the transcriptome data.

## 3. Discussion

The technique of grafting had been widely adopted in agriculture to enhance crop quality long before the underlying physiological, biochemical, or molecular mechanisms involved were comprehended [35]. In recent years, grafting onto appropriate rootstocks has been proven to enhance scions’ resistance against biotic and abiotic stresses in horticultural crops, such as bell pepper (*Capsicum annuum*) [36], chrysanthemum (*Dendranthema morifolium*) [37], melon (*Cucumis melo*) [38], apricot plants (*Prunus armeniaca*) [39] and rose [29]. Grafting has become popular in production practice of Chinese hickory recently because of its advantages in shortening plant height and decreasing juvenile phase duration [40]. It is also reported that grafting onto *C. hunanensis* developed resistance against *B. dothidea* trunk canker disease in Chinese hickory [21]. Our findings revealed that grafting onto *C. hunanensis* and *C. illinoinensis* showed better photosynthetic parameters, more stable hormone levels, and more flexible gene expression patterns under high-temperatures stress.

### 3.1. Grafting onto C. hunanensis and C. illinoinensis Enhances Photosynthesis of Chinese Hickory under HS

HS has negative effects on plant physiological processes, among which photosynthesis is highly sensitive to temperature [41]. When exposed to HS, the rate of photosynthesis is significantly reduced and heat-tolerant crop varieties exhibit higher photosynthetic efficiency compared to heat-sensitive varieties [42]. Furthermore, Mathur et al. [41] proposed that rootstock genotypes can influence the adaptability of shoots to HS in different plant species. For example, Xu et al. [43] reported that the Pn of self-grafted cucumber plants was 0.64 times lower than that of *Momordica*-grafted plants under HS conditions. In this study, the decreases in Pn in CC/CC and CC were greater than those in CC/CH and CC/CI under HS (Figure 1A), which indicated that heterologous grafted Chinese hickory (CC/CH and CC/CI) has higher heat resistance than self-grafted and non-grafted ones. Gs significantly decrease in CC and CC/CC both at 35 °C and 40 °C, while CC/CH and CC/CI decrease significantly at 40 °C (Figure 1B). Similar results were found in *Quercus suber* leaves [44], grapevine variety *Kékfrankos* [45], *Eucalyptus haemastoma* leaves [46], as exposure to high temperatures led to a marked reduction in Gs. Meanwhile, the Ci of CC and CC/CC increased markedly compared to that of CC/CH and CC/CI, indicating the inhibition of HS on the decrease in the assimilation of CO_2_ in carbon reaction. In CC and CC/CC, it is more obvious than in CC/CH and CC/CI.

Photosynthetic apparatuses such as PSI and PSII, the cytochrome b6f (Cytb6f) complex, and Rubisco can be directly harmed by HS [47]. Additionally, the inactivation of these photosynthetic apparatuses under high temperatures can inhibit various redox and metabolic reactions and disrupts photosynthetic electron transfer [41,48]. In addition, excessive light energy leads to photoinhibition and subsequent production of reactive oxygen species. Fv/Fm is a good indicator of the degree of the photosynthesis photoinhibition [49]. The decrease in Fv/Fm in CC/CH and CC/CI was smaller under HS (Figure 1D), indicating that the degree of photosynthesis photoinhibition in CC/CH and CC/CI was lower than that in CC and CC/CC. In this study, qP, Fv’/Fm’ and Φ_PSII_ showed a decreasing trend (Figure 2A–C), indicating that HS restricted the photosynthetic electron transport of plants, resulting in the hindered synthesis of ATP and NADPH, and in a decrease in the assimilation of CO_2_ in carbon reaction. The qP, Fv’/Fm’, Φ_PSII_ and NPQ of CC/CH and CC/CI showed a more stable performance, indicating that CC/CH and CC/CI could maintain a higher number of open PSII reaction centers and efficiency of light energy transformation by PSII, less heat dissipation, and linear electron transport rate than CC and CC/CC. From our results, the photosynthesis system of CC/CH and CC/CI exhibited better heat resistance under HS.

In addition, HS always disturbs the expression of genes in plants, especially those related to photosynthesis. Overall, a significant downregulation was observed in most DEGs, such as PSAD, PSAG, PET, LHCA5 and LHCA6, which are involved in photosynthetic antenna proteins, PS I and PS II complexes, and photosynthetic electron transport chain components, including F-type ATPase and cytochrome b6f complex (Figure 6A). Downregulation of these gene expressions also corresponded to the decrease in net photosynthetic rate and the decrease in Fv/Fm. Moreover, the findings suggest that HS inhibits photosynthetic electron transport, and photophosphorylation. This aligns with a previous study demonstrating the downregulation of most genes involved in photosynthesis and suppression of the photosynthetic machinery in *P. tomentosa* at 45 °C [50].

In conclusion, grafting onto *C. hunanensis* and *C. illinoinensis* improved the heat resistance of Chinese hickory by alleviating the damage of photosynthetic organs caused by HS, enhancing the photosynthetic electron transfer and photochemical activity of PSIIunder HS.

### 3.2. Grafting onto C. hunanensis and C. illinoinensis Stabilized Phytohormones in Chinese Hickory under HS

Through changes in their content, stability, activity, and transportation, hormones play key roles in enabling plants to adapt to adverse environments [3]. IAA is the most abundant auxin in plants and plays a prominent role in plant adaptation to environmental stresses, including HS [51]. Previous studies have shown that the content of IAA decreased under high-temperature stress. For example, during HS, the levels of IAA decreased by 20.93% in *Ginkgo biloba* L. [52], leading to a drastic restriction in heat-induced growth in auxin signaling in mutants or transgenic plants with lower IAA levels [53]. In our study, though IAA contents reduced in all four hickory groups under 40 °C, these were higher in CC/CH and CC/CI than in CC and CC/CC, which helped CC/CH and CC/CI maintain higher heat resistance. Similar results were reported in rice [54], where tolerant genotypes maintained high levels of IAA compared with susceptible genotypes. From our transcriptome results, lower expression of IAA synthesis genes, i.e., TAA/TRAs and YUC, and higher expression of genes related to IAA deactivation and degradation, i.e., GH3 and DAO, result in higher IAA content in CC/CH and CC/CI compared to CC and CC/CC under HS. The better IAA homeostasis helped Chinese hickory regulate in plant reproduction development, senescence, abscission, etc., under HS and adapt to HS better.

In general, ABA can synergistically interact with other phytohormones to facilitate plants’ adaptation to abiotic stress [15]. Recent studies have indicated that ABA enhances both basal and acquired thermotolerance by improving the efficiency of photosystem II (PSII) and preventing photoinhibition, minimizing the detrimental effects on the chloroplast ultrastructure [55]. Li et al. [56] reported that greater increases were found in the ABA contents of heat-tolerant plants than in WT plants under HS conditions in rice. Moreover, it has been reported that heat-induced damage is more severe in ABA-deficient mutants than in their parental cultivars [57]. Therefore, the enhanced accumulation of ABA content in response to heat stress significantly contributed to an improved thermotolerance. In this study, the gene expression of ZEP and NCED, key enzymes in ABA biosynthesis, increased in all four Chinese hickory groups, while the expression was higher in CC/CH and CC/CI than in CC and CC/CC (Figure 7B). And this was consistent with the changes in ABA content (Figure 3B). Moreover, the percentage of ABA increase in CC/CH and CC/CI exceeded 100%, surpassing that observed in CC and CC/CC (Figure 3B).

The involvement of other hormones, such as CTK, and JA, has also been documented as playing pivotal roles in plant heat stress response. HS can inhibit the synthesis and transport of CTKs, leading to a reduction in CTK content in both shoots and roots [58]. Studies have demonstrated a significant decrease in CTK levels under heat stress conditions in rice, loquat, *Arabidopsis*, and *Phalaenopsis* [59,60,61]. In comparison to the heat-susceptible varieties, the heat-tolerant rice variety SY63 exhibited a lesser reduction in panicle CTK abundance and stable CKX activity under high-temperature treatments [60]. In this study, the expression of *ipt* genes was found to be downregulated in all four Chinese hickory groups under HS (Figure 7C). This also consistent with the endogenous CTK content detected in our study (Figure 3C). JAs can effectively enhance plant resistance to HS by modulating the expression of key HSF genes such as *WRKY40*, *bZIP3*, *BHLH114*, *BHLH137*, and *WRKY8* [11,62,63,64]. JA content decreased significantly with the increase in stress temperature. This also corresponds to the results of the transcriptome that most genes downregulated under HS.

In our study, the content of plant hormones in Chinese hickory grafted onto *C. hunanensis* and *C. illinoinensis* changed less, which helped the plants maintain higher heat resistance.

### 3.3. Grafting onto C. hunanensis and C. illinoinensis Activated More Transcription Factors and Heat Shock Proteins in Chinese Hickory under HS

Besides the genes related to photosynthesis and plant hormones, transcription factors, which have been identified in at least 64 plant families and play crucial roles in enhancing plant defense mechanisms and pivotal biological processes [65], were found to be differentially expressed. In this study, differentially expressed TF genes were classified into 50 TF families. Among these, HSFs emerged as the most significant family associated with HS; the degree of upregulation for most HSF genes was higher in CC/CH and CC/CI compared to CC and CC/CC (Figure 6C). Furthermore, HSP genes were activated by HS and we observed that most of the differentially expressed HSP were small HSPs (sHSPs) (Figure 6B). Functioning as ATP-independent companions, sHSPs play positive roles in enhancing plant stress adaptation and protecting photosystem II from stress-induced damage [3,66]. The expression levels of many sHSPs, including *HSP 22*, *HSP17.4A*, were higher in CC/CH and CC/CI compared to CC and CC/CC (Figure 6B). This may contribute to the greater heat tolerance of CC/CH and CC/CI.

## 4. Materials and Methods

### 4.1. Plant Materials and High-Temperature Treatment

Three grafted Chinese hickory groups, with scions derived from *C. cathayensis* (CC) grafted onto *C. hunanensis*, *C. illinoinensis*, and *C. cathayensis,* and one non-grafted *C. cathayensis* were used in this study. These grafted groups were labeled as CC/CH, CC/CI, CC/CC and CC, respectively. The rootstocks used were two-year-old unspecified seed trees except for *C. illinoinensis*, referred to here as “Pawnee”. After 1 year of grafting, 15 plants of each group with similar growth state were subsequently transferred to artificial climate incubators (GTOP-500D, Zhejiang Topu Instrument, Ningbo, China) and pre-cultured for 1 month under controlled conditions: white light intensity of 600 µmol·m^−2^·s^−1^, relative humidity of 70%, and temperature maintained at 25 °C/20 °C (14 h day/10 h night). The high-temperature treatments were conducted in artificial climate incubators. Each grafting group was divided into three subgroups (5 plants each): one subjected to high-temperature treatment (35 °C/25 °C; 14 h day/10 h night), another exposed to severe high-temperature treatment (40 °C/35 °C; 14 h day/10 h night), while the last subgroup served as the control group with a constant temperature of 25 °C/20 °C (14 h day/10 h night). After four days of high-temperature treatment in artificial climate incubators, three biological replicates consisting of randomly selected leaves from each grafting group under different treatments were frozen in liquid nitrogen and stored at −80 °C for further use.

### 4.2. Determination of Gas Exchange and Chlorophyll Fluorescence Parameters

The Li-6400 portable photosynthetic measurement system (LI-COR, Lincoln, NE, USA) was utilized to measure gas exchange parameters. The leaf chamber was equipped with an integrated leaf chamber fluorometer (6400-40). The leaf chamber temperature was set at 25 °C. The photosynthetic active radiation (PAR) intensity was set at 1200 µmol·m^−2^·s^−1^, while the CO_2_ concentration remained constant at 400 µmol·mol^−1^. Carbon-dioxide-filled small steel cylinders were employed in the Li-6400 to maintain stable gas concentrations. The first 1–2 fully unfolded leaves from the parietal leaf of the new tip of the plant were selected. Three plants were randomly selected for each treatment and each leaf was repeatedly measured 3 times. Changes in net photosynthetic (Pn), stomatal conductance (Gs), and intercellular CO_2_ concentration (Ci) were determined. In addition, fluorescence parameters, such as maximum efficiency of PSII (Fv⁄Fm), effective quantum yield of PSIi photochemistry (Fv’/Fm’), the efficiency of PSII (Φ_PSII_), photochemical (qP), and nonphotochemical quenching (NPQ), were determined synchronously by Li-6400.

### 4.3. Phytohormone Extraction and Ultra-High-Performance Liquid Chromatography

After 4 days of treatment at elevated temperatures, about 5 cm shoots from the plant roots in each experimental group were carefully harvested and subsequently cleaned with deionized water for hormone measurements before being frozen in liquid nitrogen. Three samples were taken for each treatment group. The samples took from the same trees were used for photosynthetic determinations. The endogenous plant hormones were determined by Wuhan Greensword Creation Technology Co. Ltd., (Wuhan, China) (http://www.greenswordcreation.com/ (accessed on 12 July 2024)) based on UHPLC-MS/MS analysis (Thermo Scientific Ultimate 3000 UHPLC coupled with TSQ Quantiva, Waltham, MA, USA). The detection method followed a method previously established by Cai et al. [67]. Quantification of endogenous GAs was conducted as described previously [68].

### 4.4. RNA Extraction, cDNA Library Preparation, and Sequencing

The total RNA of Chinese hickory plant leaves was extracted using TRIzol reagent (Invitrogen, Carlsbad, CA, USA) after high-temperature treatment. Prior to constructing DNA libraries, the purity and integrity of the RNA were assessed. mRNA was enriched and fragmented into short fragments for first-strand cDNA synthesis. Subsequently, second-strand cDNA was synthesized using DNA Polymerase I and adaptor sequences were ligated to the cDNA. The Illumina HiSeq 2000 sequencing platform was employed for paired-end sequencing analysis (2 × 150 bp) of the DNA libraries.

### 4.5. Transcriptome Sequencing (RNA-seq) Data Processing and Analysis

The Fastp software (0.12.4) package was utilized with default parameters to remove reads containing adapters, low-quality bases, and undetermined bases, ensuring the acquisition of clean data. Subsequently, the clean reads were aligned to the reference genome using HISAT2. The link for the reference genome is https://www.ncbi.nlm.nih.gov/bioproject/PRJNA435846/ (accessed on 15 July 2023). Differentially expressed genes (DEGs) were determined based on thresholds of |log2 (fold change)| ≥ 1 and *p*-value < 0.05. Enrichment analyses for gene ontology (GO) (http://www.geneontology.org/ (accessed on 15 July 2023)) and Kyoto Encyclopedia of Genes and Genomes (KEGG) (https://www.kegg.jp/kegg/ (accessed on 15 July 2023)) were conducted among all DEGs.

### 4.6. Quantitative Real-Time PCR

To validate gene expression, real-time PCR was performed on randomly selected genes. Total cellular RNA was purified using Trizol RNA reagent (Life Technologies, Carlsbad, CA, USA) according to the manufacturer’s instructions. qRT-PCR analysis was conducted using the PrimeScript^TM^ RT reagent kit with gDNA Eraser (perfect real-time; Code No. RR047; Takara, Shiga, Japan) following the manufacturer’s protocol. Three technical replicates were carried out for each sample in each of the three biological repeats using *CcActin* as an internal control. The primers were designed with Primer 5.0 and are listed in Table S1.

### 4.7. Data Analysis

The data were subjected to two-way analysis of variance (ANOVA), followed by Duncan’s pairwise comparison test, for statistical analysis. Significant differences at the 5% confidence level (*p* < 0.05) are indicated by different lowercase letters, while nonsignificant differences are represented by the same lowercase letters. Statistical analysis was performed using SPSS 18.0 (IBM Corp, Armonk, NY, USA). A one-way analysis of variance (ANOVA) was conducted to analyze the transcriptome results and identify significantly differentially expressed genes (DEGs) with *p*-values < 0.05.

## 5. Conclusions

The present study aims to investigate the impact of grafting onto different rootstocks on the thermal tolerance of Chinese hickory. Our results showed that CC/CH and CC/CI exhibited superior stability in gas exchange parameters, elevated photosynthesis efficiency, and attenuated alterations in phytohormonal levels compared to CC/CC and CC. Furthermore, these grafts display a more pronounced and adaptive transcriptional response to heat stress (HS), characterized by a greater abundance of differentially expressed genes (DEGs) that positively contribute to HS tolerance. Specifically, genes involved in photosynthesis (*PSAD*, *PSAG*, *PET*, *LHCA5*, *LHCA6*) and phytohormone synthesis/metabolism (*ARF*, *GH3*, *PYL*, *PP2C*, *ABF*) play crucial roles in mediating the HS response in CC/CH and CC/CI grafts. Consequently, grafting onto compatible rootstocks enhances the heat resistance of Chinese hickory by bolstering photosynthesis and stabilizing phytohormone homeostasis. This study lays the groundwork for the selection of optimal rootstocks to improve the heat tolerance of *C. cathayensis*. Further exploration of the molecular mechanisms underlying the positive influence of rootstocks on scion heat tolerance and the role of plant hormones in mitigating heat stress is essential to deepen our understanding of heat tolerance in Chinese hickory and other grafted plant species.

## Figures and Tables

**Table 1:** Numbers of DEGs in different comparable groups.

Comparation	Upregulated	Downregulated	Total
35-CC/CI vs. 25-CC/CI	1587	2104	3691
40-CC/CI vs. 25-CC/CI	3324	3563	6887
40-CC/CC vs. 25-CC/CC	3446	2512	5958
35-CC vs. 25-CC	345	313	658
35-CC/CH vs. 25-CC/CH	2442	1374	3816
35-CC/CC vs. 25-CC/CC	1435	2066	3501
40-CC/CH vs. 25-CC/CH	3475	3583	7058
40-CC vs. 25-CC	2015	1730	3745

Note: The default parameters for screening genes with differential expression with a *p*-value < 0.05 and a fold difference greater than 2. Numbers 25, 35 and 40 indicate grafted plants treated at 25 °C, 35 °C and 40 °C for 4 days, respectively.

## Data Availability

Data availability statements are present in the “RNA-Seq Analysis” section. All reads used in this study were deposited in the NCBI Sequence Read Archive under accession number PRJNA989518.

## Supplementary Materials

The following supporting information can be downloaded at: https://www.mdpi.com/article/10.3390/plants13141967/s1, Figure S1: Principal component analysis of the transcriptome data. CC: non-grafted *Carya cathayensis*, CC/CC: self-grafted *Carya cathayensis*, CC/CH: *Carya cathayensis* grafted onto *Carya hunanensis*. CC/CI: *Carya cathayensis* grafted onto *Carya illinoinensis*. Numbers 25, 35 and 40 indicate grafted plants treated with 25 °C, 35 °C and 40 °C for 4 days, respectively. Figure S2: Correlation analysis of samples. CC: non-grafted *Carya cathayensis*, CC/CC: self-grafted *Carya cathayensis*, CC/CH: *Carya cathayensis* grafted onto *Carya hunanensis*. CC/CI: *Carya cathayensis* grafted onto *Carya illinoinensis*. Numbers 25, 35 and 40 indicate grafted plants treated with 25 °C, 35 °C and 40 °C for 4 days, respectively. Figure S3: The top 10 enriched GO terms of the three GO categories in four grated *Carya cathayensis* comparisons after 35 °C treatments. BP: biological process; CC: component category; MF: molecular function. (A): 25-CC vs. 35-CC, (B): 25-CC/CC vs. 35-CC/CC, (C): 25-CC/CH vs. 35-CC/CH, (D): 25-CC/CI vs. 35-CC/CI; Numbers 25 and 35 indicate grafted plants treated with 25 °C and 35 °C for 4 days, respectively. CC: non-grafted *Carya cathayensis*, CC/CC: self-grafted *Carya cathayensis*, CC/CH: *Carya cathayensis* grafted onto *Carya hunanensis*. CC/CI: *Carya cathayensis* grafted onto *Carya illinoinensis*. The default parameters for screening genes with differential expression with a *p*-value < 0.05 and a fold difference greater than 2. Figure S4: The top 20 enriched KEGG pathways in four grafted *Carya cathayensis* comparisons after 35 °C treatments. (A): 25-CC vs. 35-CC, (B): 25-CC/CC vs. 35-CC/CC, (C): 25-CC/CH vs. 35-CC/CH, (D): 25-CC/CI vs. 35-CC/CI; 25 and 35 indicated grafted plants treated with 25 °C and 35 °C for 4 days, respectively. CC: non-grafted *Carya cathayensis*, CC/CC: self-grafted *Carya cathayensis*, CC/CH: *Carya cathayensis* grafted onto *Carya hunanensis*. CC/CI: *Carya cathayensis* grafted onto *Carya illinoinensis*. The default parameters for screening genes with differential expression with a *p*-value < 0.05 and a fold difference greater than 2. Table S1: Gene-specific primers for qRT-PCR; Table S2: Summary of the transcriptome results; Table S3: Top transcription factor families with the most differentially expressed genes in four grafted *Carya cathayensis* comparisons under 40 °C heat stress; Table S4: Details of the top 10 enriched GO terms of the three GO categories; Table S5: Details of the top 20 enriched terms of KEGG; Table S6: DEGs related to photosynthesis, hormone transduction, HSP and HSF under 35 °C treatments compare to control.
