# Supplementary figures and images for: Two Carya Species, Carya hunanensis and Carya illinoinensis, Used as Rootstocks Point to Improvements in the Heat Resistance of Carya cathayensis
Source: Plants (Basel). 2024 Jul 18;13(14):1967. doi: 10.3390/plants13141967 (PMC11281051; doi:10.3390/plants13141967)

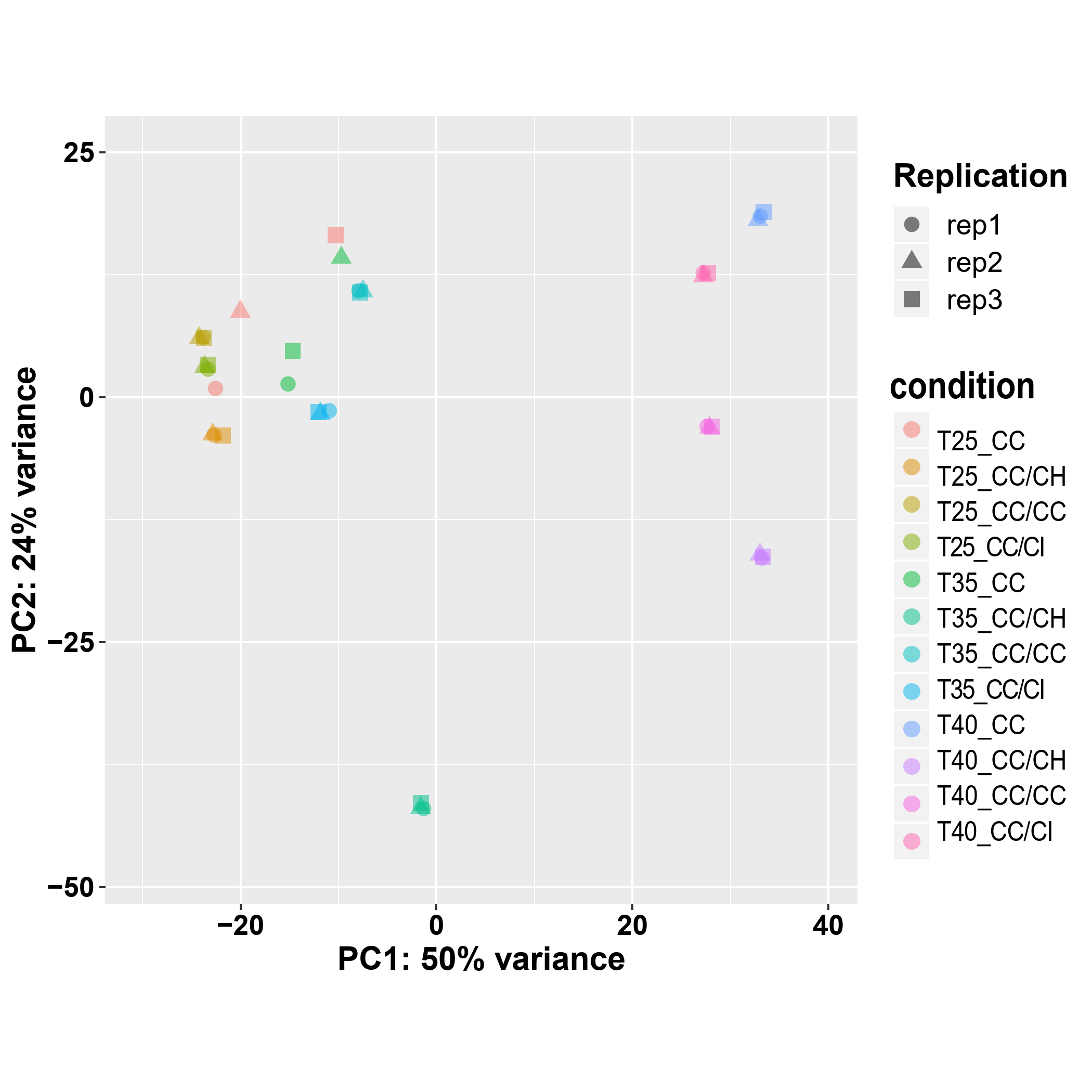

Supplement: Supplementary file 1 [file plants-13-01967-s001.zip › Figure S1.tif]
